# Comparison of the effect of melatonin, dexmedetomidine, and gabapentin on reduction of postoperative pain and anxiety following laminectomy: a randomized clinical trial

**DOI:** 10.1186/s12871-022-01851-x

**Published:** 2022-10-15

**Authors:** Reza Jouybar, Somayeh Kazemifar, Naeimehossadat Asmarian, Ali Karami, Saeed Khademi

**Affiliations:** Anesthesiology and Critical Care Research Center, Shiraz University of Medical Sciences, Shiraz, Iran

**Keywords:** Melatonin, Dexmedetomidine, Gabapentin, Pain, Anxiety, Laminectomy, Sedation, Analgesics

## Abstract

**Background:**

This study aimed to compare the effects of melatonin, dexmedetomidine, and gabapentin on postoperative pain and anxiety following laminectomy.

**Methods:**

In this randomized clinical trial, 99 patients aged 40–60 years old with American Society of Anesthesiologists physical status I-II undergoing laminectomy were divided into three groups receiving 600mg gabapentin (group G), 10mg melatonin (group M), or starch tablets (group D). The Hospital Anxiety and Depression Scale (HADS) was used to measure postoperative anxiety while a Visual Analogue Scale (VAS) was employed to measure pain severity. Patients’ satisfaction with pain treatment was also measured together with the frequency of nausea and vomiting.

**Results:**

The postoperative HADS decreased in all groups over time. Time and group had no significant interaction effect on the HADS score. Patients in the melatonin group had lower HADS at 2 and 6h after surgery. According to the VAS, the groups significantly differed in pain scores 6 and 24h after surgery. Lower VAS scores were observed 6h after surgery in the dexmedetomidine group compared with the gabapentin group and 24h after surgery in the dexmedetomidine group compared with the gabapentin and melatonin groups. Narcotic requirements, patients’ satisfaction, and vital sign changes did not significantly vary among the groups. Notably, patients in the melatonin group had less nausea and vomiting.

**Trial registration:**

This study was registered in the Iranian Registry of Clinical Trials (No. IRCT20141009019470N82, 29.06.2019) where the trial protocol could be accessed.

**Conclusion:**

Melatonin is effective as a postoperative anti-anxiety drug. Dexmedetomidine is useful in reducing postoperative pain.

## Introduction

Spinal procedures are associated with intense pain in the postoperative period, mostly in the first few days after surgery. Effective pain management leads to improved functional outcomes, early discharge, early ambulation, and prevention of chronic pain [1]. The activation of various pain mechanisms such as nociceptive, neuropathic, and inflammatory pathways may result in postoperative pain [2]. Back pain originates from diverse tissues such as vertebrae, ligaments, nerve root sleeves, dura, facet joint capsules, fascia, intervertebral discs, and muscles. Various nociceptors and mechanoreceptors are capable of stimulating pain transmission. Back pain is mostly localized in subjects when the referred pain persists in the postoperative period [3]. The intensity of postoperative pain is directly related to the number of vertebrae involved in the surgery [4]. The site of the surgery does not seem to have any effect on pain severity, which remains similar across lumbar spine, thoracic, and cervical operations [5, 6].

Various medications such as morphine (delivered by an intravenous patient-controlled analgesia pump), fentanyl (continuous injection through an epidural catheter), N-methyl-D-aspartate (NMDA) receptor antagonists, corticosteroids, nonsteroidal anti-inflammatory drugs (NSAIDs), clonidine, capsaicin, and tapentadol are used to relieve post-laminectomy pain. The mechanism of each of the above drugs is different and they have several side effects such as nausea, vomiting, platelet dysfunction, hemorrhage, gastric ulceration, renal toxicity, and respiratory depression [1, 7–9].

Dexmedetomidine is a selective α2 adrenergic receptor agonist that is metabolized in the liver and is primarily excreted via the kidneys. Dexmedetomidine facilitates arousable sedation with no effect on the respiratory drive; it also reduces the need for inhaled anesthetics. However, due to hemodynamic changes, the drug should be used carefully for conscious sedation (CS), monitored anesthetic care (MAC), or general anesthesia. [9–14]. Despite being an imidazole compound, dexmedetomidine possesses analgesic effects and minimizes postoperative opioid consumption as well as nausea, vomiting, and anxiety [15, 16].

Melatonin is mainly secreted from the pineal gland by the suprachiasmatic nucleus. This neurohormone possesses a circadian secretion pattern and regulates the biological clock; it also offers antiemetic, analgesic, and anxiolytic effects. Due to its effect on both acute and chronic pain, melatonin fulfills a beneficial role in reducing postoperative opioid consumption while minimizing nausea and vomiting. In addition, melatonin can be used to moderate the effect of light on the autonomic system [15, 17–20].

Several studies have reported that melatonin, as an analgesic, anti-inflammatory, anxiolytic, and anti-agitation premedication, is associated with sedation and anxiolysis without adverse effects on recall and driving performance. Furthermore, some studies have found confirmed the effects of melatonin on pain and anxiety, though a consensus is yet to be reached in this regard [21, 22].

Gabapentin is an anticonvulsant drug classified under the category of gabapentinoids, which are prescribed for diabetic and post-herpetic neuralgia (PHN) patients. Preemptive use of gabapentin reduces the severity of postoperative pain, thereby minimizing the use of analgesics such as narcotics. On the other hand, gabapentin reduces postoperative stress, anxiety, nausea, and vomiting. The amount of anesthetic required for general anesthesia is also reduced when gabapentin is administered [18, 23–25].

Based on the mentioned data, the present study aimed to compare the effects of melatonin, dexmedetomidine, and gabapentin on post-laminectomy pain, and anxiety.

## Materials and methods

After procurement of the institutional ethics committee’s approval and registration with the Iranian Registry of Clinical Trials (No. IRCT20141009019470N82, 29.06.2019), written informed consent was obtained from all patients who met the inclusion criteria at Abu Ali Sina Hospital (a teaching hospital affiliated to Shiraz University of Medical Sciences, Shiraz, Iran). We performed the study from August 2019 to November 2019. Ninety-nine patients aged 40–60 years old with American Society of Anesthesiologists (ASA) physical status I-II scheduled for laminectomy at one or two levels were recruited.

Patients with a history of allergic reactions to melatonin, dexmedetomidine, and gabapentin ; history of cardiovascular disease (heart failure, heart block, or bradycardia); history of kidney disease (plasma creatinine level > 1.5mg/dl or glomerular filtration rate < 50 ml/min/1.73m^2^); liver disease (aspartate transaminase, alanine transaminase, and bilirubin levels more than twice the upper limit of normal); psychological and cognitive disorders; dementia; major depression; circadian rhythm disorders such as chronic fatigue syndrome and drowsiness; or patients who had difficulty in communication were excluded from the study.

## Anesthesia technique:

After the patients’ arrived in the operating room, routine monitoring such as non-invasive blood pressure measurement, heart rate monitoring, electrocardiogram, and pulse oximetry was done. Since our patients avoided drinking water for 2–3h before entering the operating room, they were slightly dehydrated. Therefore, all patients were injected with approximately 10 ml/kg of isotonic fluid based on the history-wise and clinical conditions. Midazolam (0.04mg/kg), fentanyl (2 mic/kg), and morphine (0.1mg/kg) were injected as premedication after entering to operation room as a routine of our experiences. Anesthesia was induced using propofol (1.5-2mg/kg) and cis-atracurium (0.15mg/kg). Intubation was performed with size 7 (women) or 8 (men) endotracheal tubes. Isoflurane and nitrous oxide-oxygen (50% /50%) were used for maintenance of anesthesia (1.2–2.5%) and repeated cis-atracurium for muscle relaxation during operation based on clinical judgment and monitoring.

## Randomization and interventions:

The sample size was calculated based on a study conducted by Cheghazardi et al. [18], which showed a difference in the overall mean Visual Analogue Scale (VAS) score between patients receiving dexmedetomidine (4.80 ± 1.00) and gabapentin (4.08 ± 1.03) and determined an optimal sample size of 33 patients (without dropout) in each group to provide a power of 80% with an alpha error of 0.05. Since the current study had three groups, 33 patients were assigned to each group using block randomization with 4, 6, and 8 blocks. The list of the blocks was extracted from www.sealedenvelope.com, with both the investigators and patients being kept blind to the size of the blocks. Before the study, 99 closed envelopes were prepared to contain oral tablets in non-specified boxes. Each envelope had a specific number provided by a pharmacist who was blinded to the study groups. Two hours before the operation, a nurse randomly selected one of the envelopes and gave the pills to the patient.

In the melatonin group (M), 10mg (two 5mg tablets) of melatonin (Natrol, USA) were given to the patients, while in the dexmedetomidine group (D), starch tablets, and in the gabapentin group (G), 600mg (two 300mg tablets) of gabapentin (RAZAK, Iran) were administered.

To deliver the dexmedetomidine (PFIZER, USA) while keeping the patients blinded, a nurse received a 20 ml syringe from a second nurse according to the patients’ code and weight. In the melatonin and gabapentin groups, the syringe contained normal saline, while in the dexmedetomidine group, it contained a diluted solution of dexmedetomidine (1µg/kg). All three syringes contained 20 ml of clear solution and were injected in the prone position within ten minutes after induction of anesthesia. Information was recorded by another nurse during the operation using forms included in the envelopes. The patients and nurses were unaware of the type of prescription drugs and the nature of the study group.

## Assessments:

All patients were visited by an anesthesiologist the night before surgery; a medical history was taken and a clinical examination was performed. Also, a green mark was placed on the patient’s file if he/she met the inclusion criteria and entered the study.

In this study, we compared the effects of melatonin, dexmedetomidine, and gabapentin on the primary outcomes of postoperative pain and anxiety and the secondary outcomes of the occurrence of nausea and vomiting, required amount of analgesics after surgery, patients’ satisfaction, and hemodynamic changes. Psychological tests were validated for the Iranian study populations used in this study.

To measure the severity of pain, a VAS was used; the patients’ pain levels were measured at 2, 6, and 24h after surgery. The total consumption of analgesics (morphine, acetaminophen, and meperidine) was also recorded. In the recovery room, the patients were trained to inform the nurses whenever their pain score was four or higher on the VAS. At such times, the patients received an IV bolus of 0.02mg/kg morphine and were re-evaluated after 20min. If the patient’s pain score was four or above again, the same dose of morphine was injected with/under respiratory monitoring (rate and depth of breathing). In the ward, patients with pain (VAS score ≥ 4) were given an IV infusion of 1g acetaminophen (Apotel®). The patient was re-evaluated after an hour and given a 25mg meperidine injection if the pain score remained greater than or equal to four.

The Hospital Anxiety and Depression Scale (HADS) was used to measure postoperative anxiety 2, 6, and 24h after surgery [26]. Scores of zero to seven were regarded as normal, 8 to 10 signified borderline cases, and 11 to 21 indicated abnormal anxiety [26]. The patients’ satisfaction with postoperative pain treatment was measured based on a Likert scale [27]. At the time of discharge from recovery, patient satisfaction was measured on a five-point scale (Score 1: Very poor, Score 2: Poor, Score 3: Satisfactory, Score 4: Good, Score 5: Very good). All psychological tests were conducted by an expert psychologist. The occurrence of nausea (yes or no) and vomiting (yes or no) was also measured among the secondary outcomes. All data were recorded in predefined forms before being transferred in sealed envelopes to a statistician who was blinded to the study groups.

### Data Analysis

In this study, continuous variables were reported as mean and standard deviation or median and interquartile range (IQR). Categorical variables were reported as numbers and percentage. Differences between groups were examined by analysis of variance (ANOVA); the Tukey post hoc test was utilized to analyze parametric variables and the Kruskal-Wallis test was used for non-parametric variables. Also, the chi-squared test or Fisher’s exact test was applied to assess differences in categorical variables. We accessed the repeated measure ANOVA test for comparing the HADS within groups over time. Data were analyzed using SPSS 21 (IBM, USA) and P-values < 0.05 were considered statistically significant.

## Results

**Fig. 1:**
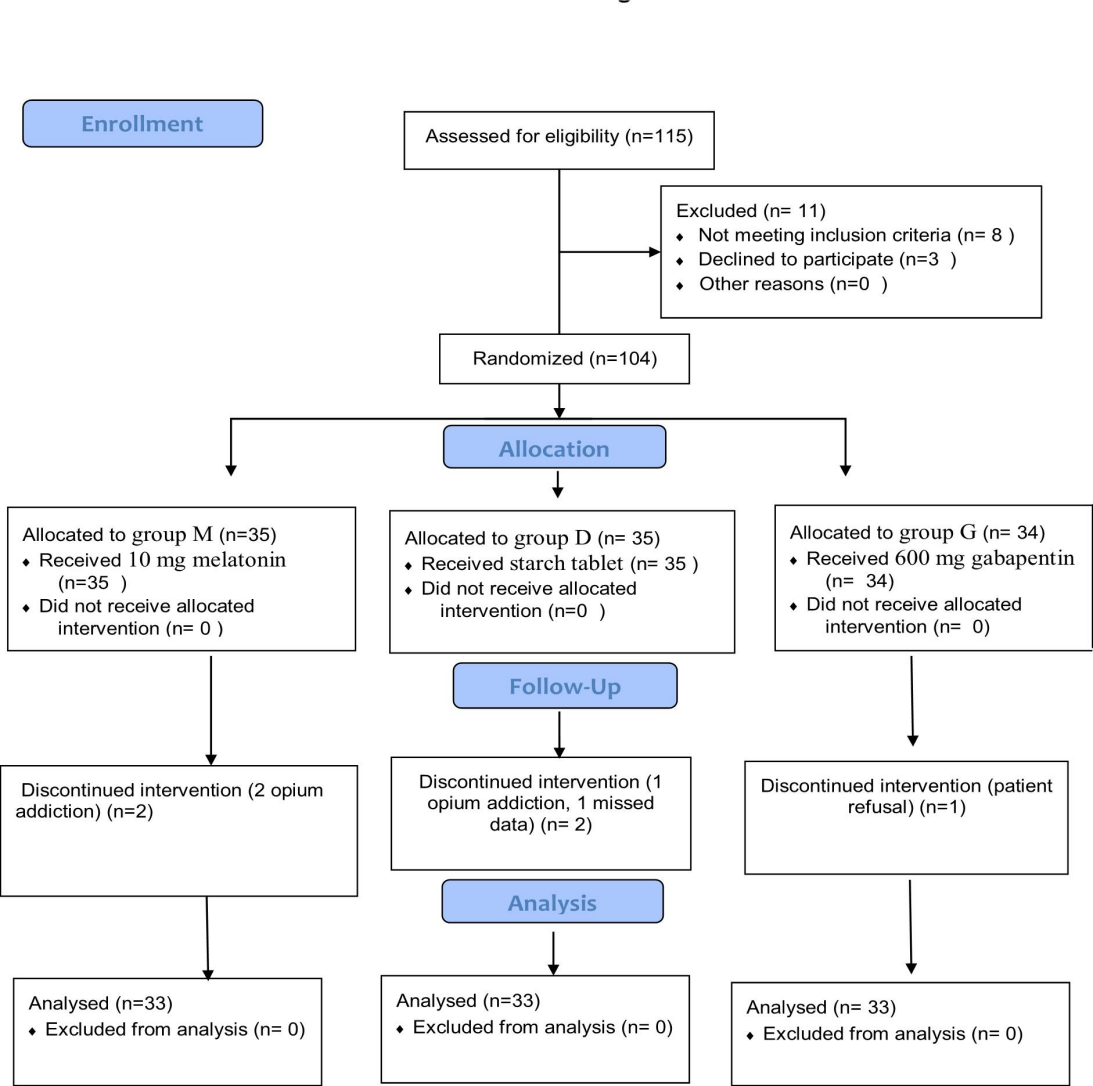
Consort Flow Diagram

In our study, 115 patients were screened for eligibility. Among them, eight were excluded due to a lack of inclusion criteria and three declined to participate. Therefore, 104 patients were enrolled and divided into three groups melatonin (N = 35), dexmedetomidine (N = 35), and gabapentin (N = 34). In the follow-up, five patients were excluded (two due to opioid intake in the melatonin group, one due to opioid intake and one due to missing data in the dexmedetomidine group, and one due to withdrawal in the gabapentin group). A total of ninety-nine patients completed the study (Fig.1).

No significant differences were found in demographic and baseline data including duration of surgery, bleeding level, recovery stay, and morphine and meperidine administration (Table1). The amount of bleeding was 230 (140–500) in the melatonin group, 200 (150–490) in the dexmedetomidine group, and 230 (150–450) in the gabapentin group (P = 0.907).

**Table 1:** Demographic characteristics and baseline data

	Melatonin	Dexmedetomidine	Gabapentin	P-value
**Age, years (mean ± SD)**	48.79 ± 9.95	47.58 ± 10.28	50.15 ± 10.55	0.596
**Sex, male (frequency; %)**	11 (33.3)	11 (33.3)	10 (30.3)	0.995
**BMI (mean ± SD)**	25.66 ± 3.6	26.12 ± 3.68	26.61 ± 3.55	0.562
**Duration of surgery, min (median; IQR)**	120 (90–150)	120 (90–120)	120 (85–120)	0.393
**Bleeding level (ml) (median; IQR)**	230 (140–500)	230 (150–450)	200 (150–490)	0.907
**Recovery time, min (median; IQR)**	120 (105–150)	120 (120–135)	110 (52–150)	0.195
**Morphine, mg(median; IQR)**	10 (10–10)	10 (10–10)	10 (8–10)	0.096
**Meperidine, mcg(median; IQR)**	150 (150–200)	150 (150–175)	150 (150–150)	0.082
Abbreviation: BMI: Body Mass Index.				

**Fig. 2:**
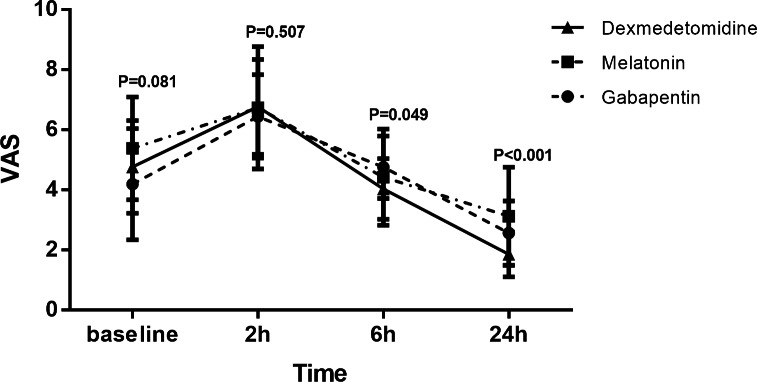
Change in pain over time in the three groups according to the Visual Analogue Scale (VAS).

At the VAS score, there was no significant difference between the groups before and two hours after surgery (P = 0.081, P = 0.507, respectively); however, significant differences were observed between the groups at 6 and 24h after surgery (P = 0.049, P < 0.0001, respectively). The patients had a lower VAS score in the dexmedetomidine group compared with the gabapentin group at 6h (P = 0.046) and the gabapentin(p = 0.018) and melatonin groups in 24h (p < 0.001) (Table2) after the operation. Due to the interaction between group and time (Fig.2), we only reported the ANOVA test.

**Table 2:** VAS scores for pain in the three intervention groups at different times

Time	Melatonin	Dexmedetomidine	Gabapentin	P-value
**Before surgery**	5.38 ± 1.71	4.76 ± 1.54	4.19 ± 1.85	0.081
**2h after surgery**	6.73 ± 2.03	6.76 ± 1.58	6.44 ± 1.39	0.507
**6h after surgery**	4.42 ± 1.60	4.03 ± 1.01*	4.75 ± 1.04	0.049
**24h after surgery**	3.12 ± 1.63	1.85 ± 0.75**	2.56 ± 1.07	< 0.0001
Scores are reported as mean ± SD. *: Dexmedetomidine is different with gabapentin (P = 0.046) **: Dexmedetomidine is different with melatonin and gabapentin (P < 0.001 and P = 0.018, respectively)				

**Fig. 3:**
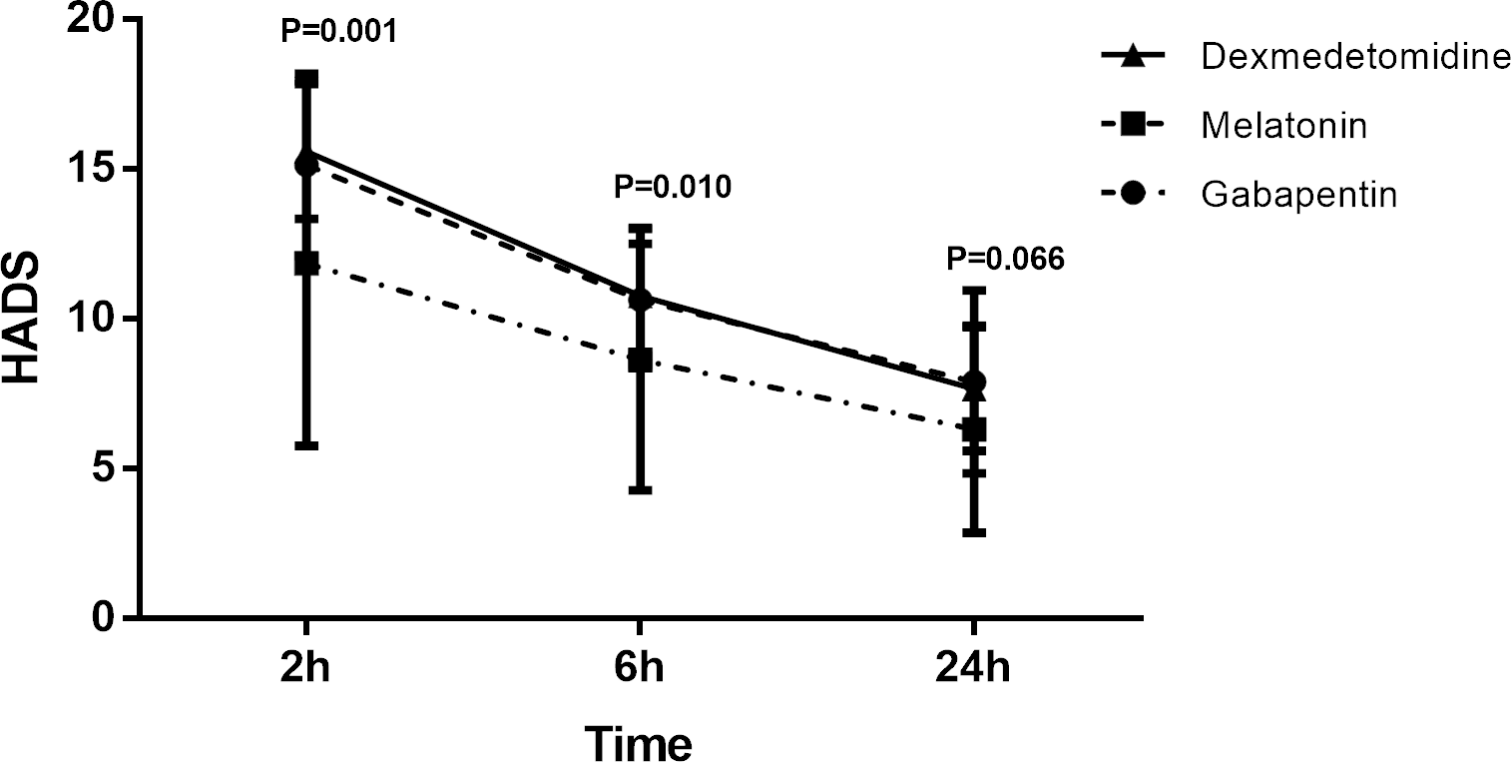
Change in anxiety over time in the three groups according to the Hospital Anxiety Depression Scale (HADS).

Postoperative HADS decreased in all groups over time (P < 0.001). Time and group did not have a significant interaction effect on the HADS score (P = 0.065). In general, the melatonin group had a lower mean HADS score compared with the other two groups (P < 0.001) (Fig.3). According to the ANOVA test, there were significant differences in HADS between the groups, two and six hours after surgery (P = 0.001 and P = 0.010, respectively). In other words, patients in the melatonin group had lower HADS compared with those in the dexmedetomidine (P = 0.001) and gabapentin (P = 0.007) groups two hours after surgery. Also, patients in the melatonin group had inferior HADS compared with the dexmedetomidine (P = 0.017) and gabapentin (P = 0.035) groups at the sixth postoperative hour (Table3).

**Table 3:** HADS in the three intervention groups at different times

Time	Melatonin	Dexmedetomidine	Gabapentin	P-value
**2h**	11.84 ± 6.09^*^	15.57 ± 2.25	15.13 ± 3.04	0.001
**6h**	8.63 ± 4.36^**^	10.75 ± 2.27	10.62 ± 1.87	0.010
**24h**	6.30 ± 3.45	7.66 ± 2.07	7.89 ± 3.05	0.066
Scores are reported as mean ± SD. *: Melatonin is different with dexmedetomidine (P = 0.001) and gabapentin (P = 0.007). **: Melatonin is different with dexmedetomidine (P = 0.017) and gabapentin (P = 0.035).				

The median change in HADS score between the second and twenty-fourth hours varied significantly between the groups (P = 0.007); there was significantly less change in the melatonin group compared with the dexmedetomidine (P = 0.04) and gabapentin (P = 0.01) groups. A significant difference also existed in the median change in HADS score between 2 and 6h (P = 0.017), with the melatonin group having less change relative to the gabapentin group (P = 0.025). There was no significant difference between the groups in terms of median change in HADS score between 6 and 24h (P = 0.228) (Table4).

**Table 4:** Comparison of median changes in HADS over time in the three intervention groups

Time difference	Melatonin	Dexmedetomidine	Gabapentin	P-value
**2-24h**	6 (2.5–8.5) ^*^	8 (7–10)	9 (5.5–11)	0.007
**2-6h**	4 (1–5) ^**^	5 (4–6)	6 (4–7)	0.017
**6-24h**	2 (1-3.5)	3 (2–4)	4 (0.5–4.5)	0.228
Changes in scores are reported as median (IQR). *: Melatonin is different with dexmedetomidine (P = 0.04) and gabapentin (P = 0.01). **: Melatonin is different with gabapentin (P = 0.025)				

Differences in requirement of narcotics (P = 0.457), patient satisfaction (P = 0.193), and vital sign changes (P = 0.451) among the groups were not statistically significant. However, the occurrence of nausea and vomiting was significantly different among the groups (P = 0.002 and P = 0.001, respectively). In other words, patients who received melatonin had less nausea and vomiting compared with those receiving dexmedetomidine or gabapentin (Table5).

**Table 5:** Comparison of clinical variables and patient satisfaction between the three study groups

		Melatonin	Dexmedetomidine	Gabapentin	P-value
**Clinical variables**	**Narcotic requirement**	2 (1–3)	2 (2–3)	2 (2–2)	0.457
	**Nausea**	0 (0–1) ^*^	1 (1–2)	2 (0–2)	0.002
	**Vomiting**	0 (0–1) ^**^	1 (1–1)	1 (0–2)	0.001
	**Vital sign change**	0 (0–1)	0 (0–1)	0 (0-0.5)	0.451
**Patient satisfaction**	**Satisfied**	26 (78.8)	24 (72.7)	27 (81.8)	0.193
	**Neutral**	7 (21.2)	9 (27.3)	4 (12.1)	
	**Unsatisfied**	0 (0)	0 (0)	2 (6.1)	
Data are reported as median (IQR) for clinical variables and as frequency (%) for patient satisfaction. *: Melatonin is different with dexmedetomidine (P = 0.004) and gabapentin (P = 0.003). **: Melatonin is different with dexmedetomidine (P = 0.002) and gabapentin (P = 0.001).					

## Discussion:

Pain and anxiety are two pivotal postoperative complications that can affect patient satisfaction [1, 2]. According to the findings of this study, dexmedetomidine was more effective in controlling postoperative pain than the other two drugs, while anxiety occurred less among patients receiving melatonin.

The level of early postoperative anxiety was lower in the melatonin group than in the other two groups, with the rate of reduction in drug effectiveness of the drug also being lower. The results of our study showed that the level of early postoperative anxiety at 2 and 6h after surgery was lower in the melatonin group relative to the dexmedetomidine and gabapentin groups. However, 24h after the surgery, this difference persisted but lost its statistical significance. In other studies, similar findings have been reported after melatonin administration [28, 29].

A novel finding of this study was the greater efficacy of melatonin compared with dexmedetomidine and gabapentin in minimizing early postoperative anxiety. Due to the duration of the effect of melatonin (approximately 5h), as we approached the end of the first day after surgery, the effectiveness of the drug decreased. It is likely that by re-administering the drug after surgery, its effect can be sustained.

The anxiolytic effect of melatonin and gabapentin was also reported in a study by Marzieh-Beigom Khezri et al. However, in our study, 2 and 6h after surgery, the anti-anxiety effect of melatonin was significantly greater than dexmedetomidine and gabapentin, while in the mentioned study, no significant difference in postoperative anxiety was observed between patients receiving melatonin and gabapentin [30]. Anti-anxiety effects are among the features of dexmedetomidine, a specific α2 agonist. Perhaps the difference in anxiety findings between the mentioned study and ours is due to differences in dose, method, and timing of drug administration [30, 31]. In the study of Lim and colleagues on patients undergoing central neuroaxial blockade, the effect of the infusion of propofol and dexmedetomidine was compared; the researchers reported that dexmedetomidine caused a greater reduction in postoperative anxiety compared with propofol [32].

As far as we know, no other study has compared the efficacy of melatonin, dexmedetomidine, and gabapentin in terms of postoperative pain and anxiety. Here, we conclude that melatonin is more effective in reducing postoperative anxiety than the other two drugs.

In our study, the closer we got to 24h after surgery, the less pain there was in all three groups. However, what is important was the greater analgesic efficacy of dexmedetomidine at 6h relative to gabapentin (P = 0.046), and at 24h compared with both melatonin and gabapentin (P = 0.001 and P = 0.018, respectively).

The analgesic effects of each of the above drugs have been studied in several studies and the effectiveness of each in reducing postoperative pain has been proven [33]. Gabapentin is an amino acid with the structure of the gamma-aminobutyric acid (GABA) neurotransmitter. This drug can be used as a strong analgesic for reducing postoperative pain [34]. Studies have shown that gabapentin stimulates analgesia by stimulating peripheral, spinal, and supraspinal a2A-adenosine receptors [35]. In the study of Clark et al., it was observed that with the administration of 1200mg of gabapentin, the level of postoperative pain significantly fell in comparison with the control group [36]. However, in a cohort study by Jennifer Hah et al., it was found that despite its analgesic properties, gabapentin was not effective in postoperative pain control [37].

In a study by Borazan et al., the analgesic effects of melatonin after surgery were shown [38]. In Caumo’s study, preoperative melatonin administration gave rise to analgesic and anti-inflammatory effects [39]. The mechanism of action of melatonin is through binding to its plasma receptors, intracellular receptors (e.g., Calmodulin), and orphan nuclear receptors, with anti-inflammatory effects also being prominent [40]. Another possible reason for the reduction of pain through the use of melatonin is its anxiolytic effects through a neurobiological mechanism; it is hypothesized that anxiety activates the behavioral inhibition system, which prevents postoperative pain by consuming melatonin and intervening with this mechanism [41].

Dexmedetomidine exerts its analgesic effects by reducing the expression of the glucocorticoid receptor. The elevation of glucocorticoids during surgery causes an increase in the incidence of pain resistance due to anxiety. In fact, dexmedetomidine probably mitigates this destructive effect of glucocorticoids, thereby minimizing postoperative pain. In the study of Lu Li et al. on mice, the possible mechanism behind the reduction of anxiety-induced persistent pain by dexmedetomidine was a decrement in the expression of the glucocorticoid receptor [42]. On the other hand, Maze et al., in an animal study, reported that injecting high doses of dexmedetomidine reduced the secretion of cortisol, with the response of cortisol to adrenocorticotropic hormone (ACTH) being blunt for three hours after injection [43]. These investigations were performed on animals, but this effect has not been clinically evaluated in humans to date. Although our study revealed that the analgesic effects of dexmedetomidine were greater relative to the other two drugs, further clinical and laboratory studies are needed to confirm and explain this finding.

In our study, the comparison between the three drugs showed that dexmedetomidine was more effective than the other two in postoperative pain management. However, no significant difference was found in the amount of postoperative narcotic consumption between the three groups. This can probably be explained by the duration of the drugs’ action, their different mechanisms in pain control, as well as their anti-anxiety effects.

In our study, the rate of post-operative nausea and vomiting (PONV) was lower in the melatonin group than in the other two groups. In the study of Hyunjeong Kwak et al., the effects of administering a single dose of dexmedetomidine at the end of the operation on PONV were reported. However, in our study, dexmedetomidine was administered at the beginning of the operation as a single dose, meaning that a pronounced effect on postoperative PONV probably could not be observed considering the onset time and duration of the drug’s 15-minute peak effect [44]. In the study of Lafli Tanay, postoperative PONV levels were significantly lower in the melatonin group than in the vitamin C group due to decreased use of narcotics. However, since the number of narcotics used in our study was not different among the three groups, this factor could not explain the decreased PONV occurrence in the melatonin group [20]. In Esmat’s study, less nausea and vomiting were observed in the melatonin group than in the control group, which is consistent with our study [19]. A systemic review of the effects of melatonin in cancer patients confirmed its anti-nausea effects [45], but the mechanism of this effect of melatonin on PONV is yet to be elucidated. Therefore, more studies are needed to explain this phenomenon.

Our study has some limitations. If this study had been performed on larger sample size, there would probably have been more significant results in terms of the analgesic and anti-anxiety effects of the drugs. On the other hand, the use of dexmedetomidine as an infusion during the operation and oral reingestion of melatonin after surgery could have led to more pronounced results. To measure narcotic consumption after surgery, the use of a patient-controlled infusion pump may be better as it prevents bolus administration and its possible side effects while facilitating more accurate recordings.

In conclusion, melatonin is effective as a postoperative anxiolytic, while dexmedetomidine can be used to reduce postoperative pain. Furthermore, nausea and vomiting occurred less in the melatonin group compared with the dexmedetomidine and gabapentin groups. The mechanism of action behind these effects of melatonin should be investigated further.

In conclusion, melatonin is more effective than dexmedetomidine and gabapentin groups as a postoperative anxiolytic and nausea - vomiting medication. However, the mechanism of the antiemetic of melatonin should be investigated further. Dexmedetomidine in reducing postoperative pain is more effective than two others.

## Data Availability

All data will be available on request. Dr, Reza jouybar is responsible to provide data. If someone wants to request the data from this study can contact Dr. Reza Jouybar (jouybarr@gmail.com).

## Declarations

### Data Access and responsibility

The principal investigator, Reza Jouybar, had full access to all of the data in the study and takes responsibility for the integrity of the data and the accuracy of the data analysis.
